# Comparison of the PlusoptiX A16 and vision screener V100

**DOI:** 10.3389/fopht.2024.1414417

**Published:** 2024-09-26

**Authors:** Jorge Jorge, Paulo Fernandes

**Affiliations:** Clinical and Experimental Optometry Research Laboratory (CEORLab), Physics Center of Minho and Porto Universities (CF-UM-UP), School of Sciences, University of Minho, Braga, Portugal

**Keywords:** children, vision, refractive error, photorefraction, PlusoptiX, vision screener V100

## Abstract

**Clinical relevance:**

This study compares a novel photoscreening device with a previously validated one in a school-age population. It highlights a tendency of the new device to underestimate myopic spherical equivalent and overestimate hyperopic cases.

**Purpose:**

To compare the PlusoptiX A16 and Vision Screener V100 photoscreeners in a study population of school-age children.

**Methods:**

One hundred and thirty-three children, with a mean age of 6.4 ± 0.5 years, were evaluated using both the PlusoptiX A16 and Vision Screener V100 photoscreeners. The measurements were taken in random order in a room with diminished ambient lighting.

**Results:**

The mean refractive error values for the M component were 0.27 ± 0.67D (PlusoptiX A16) and 0.21 ± 0.58D (Vision Screener V100). For the J0 component, means were 0.16 ± 0.38D (PlusoptiX A16) and 0.06 ± 0.33D (Vision Screener V100) and for theJ45 component the means were 0.03 ± 0.17D (PlusoptiX A16) and 0.06 ± 0.22D (Vision Screener V100). When compared both instruments, statistically significant differences were observed for the M (p=0.017) and J0 (p=0.004) components. The agreement rates between PlusoptiX A16 and Vision Screener V100 across different refractive components were 80.5% for sphere, 82.0% for cylinder, and 40.6% for axis when considering a range of ±0.75 D for sphere and cylinder and ±25.0 degrees for cylinder axis. Simultaneously considering all three conditions, the overall agreement was 73.7%.

**Conclusion:**

The Vision Screener V100, while generally aligning well with PlusoptiX A16, tends to underestimate myopic spherical equivalent, overestimate hyperopic cases, and underestimate J0 astigmatism.

## Introduction

Early and accurate detection of refractive errors in infants and children has always been a concern for paediatric optometrists and ophthalmologists. Early identification and treatment of refractive errors, notably amblyopia, is crucial to prevent vision impairment in children. Childhood vision disorders often manifest asymptomatically, evading timely intervention. Addressing specific visual abnormalities during critical development stages is more effective than later in life (1). In recent years, many nations have endorsed comprehensive eye examinations for preschoolers as a routine practice to facilitate early identification of vision problems. Visual screening programs emerge as a cost-effective alternative to mitigate the time-consuming nature and higher costs associated with comprehensive eye examinations, effectively identifying children with visual impairments (2, 3). Automated vision screening programs, particularly those utilizing autorefractors and photoscreeners, hold significant promise for the early detection of vision problems in children. These devices, capable of operation by a diverse range of healthcare professionals, can effectively identify both refractive errors and amblyogenic risk factors when employed in large-scale screening initiatives (4, 5). The origins of photorefraction can be traced back to the 1960s, and significant advancements were made in the 1980s (6, 7). With the widespread use of digital imaging systems, photorefraction has become popular, leading to a wide range of available equipment. All have been shown to be effective in detecting refractive errors in young children, to varying degree. The PlusoptiX (PlusoptiX GmbH, Nurnberg, Germany) is one of the device most commonly used, and its effectiveness has been studied by several authors (8–11).

The Vision Screener V100 (Mediworks, Shanghai, China), is a portable photorefraction device, employed for refractive error measurement in children and adults. According to the manufacturer, by utilizing artificial intelligence, specifically deep learning, the equipment enhanced stability and robustness by improving data representativeness. Artificial intelligence applications focused on increasing the accuracy of measurements during vision screening, showcasing the potential synergy between advanced technology and vision assessment tools for comprehensive and precise evaluations in diverse age groups (12, 13). Both devices incorporate the manufacturer’s referral criteria to assist in the referral of subjects, taking into account the presence of amblyopiogenic factors.

The aim of the study was to compare the results obtained using two different photoscreeners systems in a population of early school-age children.

## Methods

This is a cross-sectional study that collected refractive data from a population of school-based children aged between 6 and 7 years old with a mean age of 6.4 ± 0.5 years. One hundred and thirty-three children were screened using the PlusoptiX A16 and the Vision Screener V100 in a randomized order, in a dedicated room with dim ambient lighting (mesopic condition) to ensure adequate pupil size for accurate measurements. The PlusoptiX photoscreener and Vision Screener V100 are both portable video-refractometers that additionally measure pupil size, interpupillary distance, and ocular alignment. These devices possess the capability to assess both eyes simultaneously, enabling the detection of strabismus in most instances, and are remarkably rapid in their administration. The employed software algorithm determines the refractive strength of the eye from the observed light crescent, which is readily visible within the pupil when using off-axis, infrared illumination.

To enhance the reliability of refractive measurements, three consecutive measurements were acquired for each eye using each device, and the average of these two measurements was recorded. Separate optometrists operated the devices to diminish inter-operator variability, and each measurement was completed within 30 seconds. The measurement procedure for both photorefraction devices was the same and was conducted in accordance with the guidelines for pediatric vision screening as outlined by the American Academy of Ophthalmology (AAO) (11, 14). Ambient luminance was adjusted to ensure that the pupil diameter, as measured with the PlusoptiX device, remained around 5 mm. Both devices, PlusoptiX and the comparison device, were operated under these same lighting conditions to ensure uniformity (15). Measurements were acquired consecutively using both devices. A brief pause of approximately 2 minutes was observed between measurements to allow the child to relax and to ensure consistent testing conditions. This pause helped minimize any potential accommodation effects that might influence the results. Both the PlusoptiX A16 and the Vision Screener V100 were placed at a distance of 1.00 meter from the child at eye level and adjusted back and forth until green circles were observed around the pupils. Binocular measurements were taken at this distance. Participants were excluded from the study if valid measurements could not be obtained with both devices. The parents or legal guardians of each child were informed about the tests by the schools, completed a questionnaire prior to the measurements, and provided informed consent before the evaluation. Children with strabismus or previous eye surgery confirmed based on both self-reported history and clinical observations such as visual acuity and binocular vision assessment, were not included in the study.

Traditional clinical representations of refractive error, including sphere, cylinder, and axis, are not adequate for quantitative analysis, and for this reason, spherocylindrical refractive results were converted into vector representations (M, J0 and J45) by Fourier analysis, as recommended by Thibos (16). In this study, statistical analysis was performed using SPSS for Windows software (version 29; SPSS, Inc.). The bias was statistically assessed as the mean of the differences compared to zero. The hypothesis of zero bias was examined by the non-parametric test Wilcoxon signed ranks test. The 95% limits of agreement (mean of the difference ± 1.96 x SD) were also calculated, as recommended by Bland and Altman (17, 18).

The study adhered to the tenets of the Declaration of Helsinki and approved by the Ethics Committee for Research in Life and Health Sciences (CEICVS) of the University of Minho (CEICVS 012/2023).

## Results

The study included 133 school-aged children, aged between 6 and 7 years, with a mean age of 6.4 ± 0.5 years. Among these participants, 76 (57.1%) were 6 years old, and 57 (42.6%) were 7 years old. Additionally, 73 (54.9%) were girls, and 60 (45.1%) were boys. Initially, refractive data were collected from both eyes, and no significant differences were detected between the left and right eyes (Wilcoxon test, p = 0.502). Subsequently, only right eye measurements were included in the final analysis.

**Figure 1:**
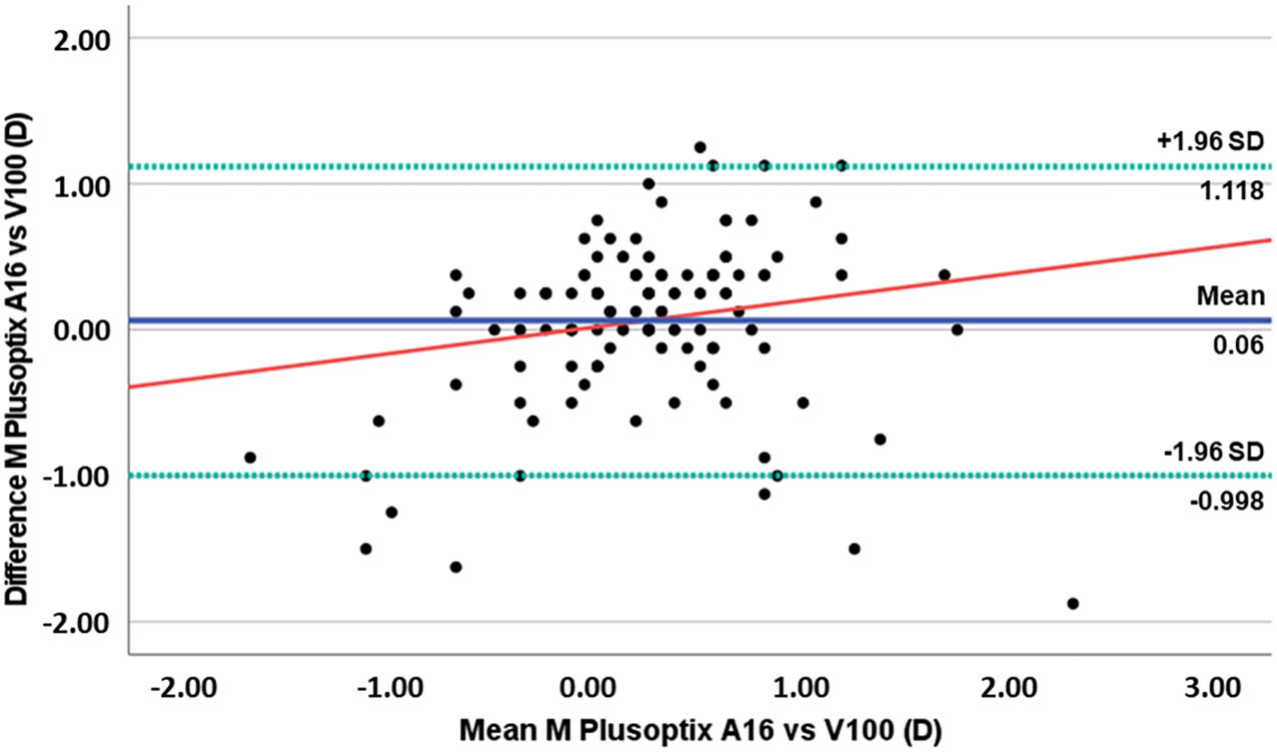
Plots of difference vs mean for M component values obtained with PlusoptiXA16 and the Vision Screener V100. (The blue solid line represents the mean bias, the green dashed lines represent 95% limits of agreement, and the red solid line represents the linear regression).

**Figure 2:**
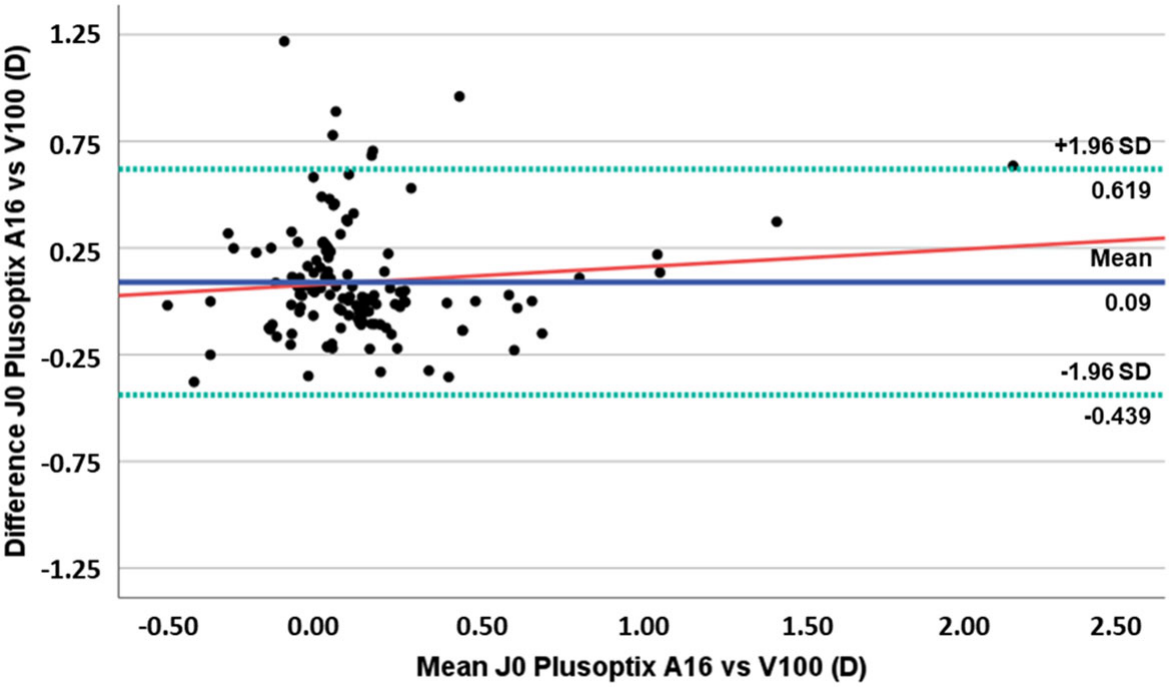
Plots of difference vs mean of J0 component values obtained with PlusoptiXA16 and the Vision Screener V100. (The blue solid line represents the mean bias, the green dashed lines represent 95% limits of agreement, and the red solid line represents the linear regression).

**Figure 3:**
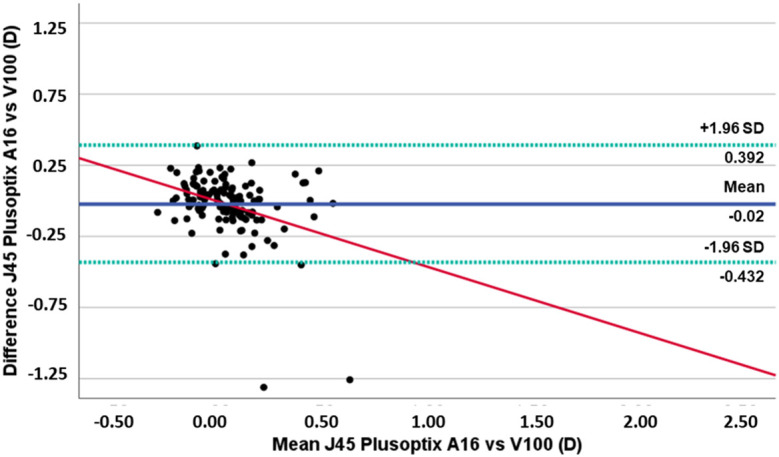
Plots of difference vs mean of J45 component values obtained with PlusoptiXA16 and the Vision Screener V100. (The blue solid line represents the mean bias, the green dashed lines represent 95% limits of agreement, and the red solid line represents the linear regression).

To visually evaluate the agreement between measurements obtained using the two devices, Bland-Altman plots representing the differences in refractive values as a function of the mean for each instrument are presented in **Figures 1**–**3** and summarized in **Table 1**. **Figure 1** displays the differences plotted as a function of the mean for the M component, indicating a slight underestimation of the spherical equivalent (M) by the Vision Screener V100 compared to the PlusoptiX A16. We also observed a tendency for the Vision Screener V100 to negatively overestimate in cases of myopia and positively overestimate in cases of hyperopia (r^2^ = 0.036, p=0.030). Importantly, this difference should not be neglected for higher levels of myopia exceeding -0.75D, where the discrepancy between the devices may have more significant clinical implications.

**Table 1:** Displays the mean and the standard deviation of the M, J0, and J45 parameters, as well as the mean difference, the level of statistical significance, and the limits of agreement at the 95% confidence interval between the two photoscreeners.

	PlusoptiX A16	Vision Screener V100	Mean difference (A16-V100)	Limits of agreement		p*
				Mean - 1.96*SD	Mean + 1.96*SD	
M (D)	0.27 ± 0.67	0.21 ± 0.58	0.06 ± 0.54	-0.998	1.118	0.017
J0 (D)	0.16 ± 0.38	0.06 ± 0.33	0.09 ± 0.27	-0.439	0.619	0.004
J45 (D)	0.03 ± 0.17	0.06 ± 0.22	-0.02 ± 0.21	-0.432	0.392	0.447

*Wilcoxon test.

**Figure 2** displays the differences in J0 component plotted against the mean for each device, depicting a slight underestimation of J0 by the Vision Screener V100 compared to the PlusoptiX A16. There was no observed trend for a systematic increase or decrease in the difference between the two devices with an increase in J0 value (r² = 0.010, p=0.265).

The differences in the J45 astigmatism component are illustrated in **Figure 3**. Although there is a slight variance in the values of the J45 component measured by the two devices, this disparity is not statistically significant. However, it is observed that as the values of the J45 component become more positive (indicating astigmatism in the 1st quadrant), there is an increased tendency to overestimation with the Vision Screener V100 (r²=0.127, p<0.001). The Bland-Altman plots reveal important clinical implications regarding the agreement between the Vision Screener V100 and PlusoptiX A16. The Vision Screener V100 tends to slightly underestimate spherical equivalent values compared to the PlusoptiX A16 and shows a tendency to overestimate myopia and underestimate hyperopia. Additionally, while it generally underestimates the J0 component without a systematic trend, it exhibits a notable tendency to overestimate J45 astigmatism values as they become more positive. Clinically, these findings suggest that while both devices are useful for refractive assessments, care should be taken when interpreting measurements, particularly for extreme cases of astigmatism, to ensure accurate diagnosis and treatment planning.

In addition to the aforementioned analyses, we evaluated the concordance between the PlusoptiX A16 and Vision Screener V100 data by calculating the differences between the measurements obtained from the two devices. Specifically, we assessed the agreement of the sphere and cylinder measurements within a range of ±0.75 D, the alignment of the cylinder axis within ±25.0 degrees, and the assessment of all three conditions simultaneously. The concordance variable was defined based on these differences: a value of 1 was assigned if the difference fell within the specified range (indicating concordance), and a value of 0 was assigned if it did not (indicating no concordance).

Regarding the sphere, we observed an agreement rate of 80.5%. For the astigmatism value, the agreement rate was 82.0%, and for the astigmatism axis, the agreement rate was 40.6%. When considering the sphere, cylinder, and axis simultaneously, the overall agreement rate was 73.7%.

This analysis revealed a mean difference of 40.0 degrees in the astigmatism axis between the PlusOptrix A16 and Vision Screener V100. Interestingly, this difference was smaller for higher astigmatisms (equal to or greater than 0.75D), where the axis difference was 33.6 degrees.

## Discussion

Early detection of visual disorders and timely treatment can effectively reduce the onset of amblyopia (19). Clinicians and researchers have long sought for easy-to-apply and highly accurate vision screening methods. The emergence of photorefraction systems has significantly advanced screening for amblyogenic factors in pediatric populations. Photo-screening technology is increasingly used due to its rapid binocular measurements, minimal training requirements, and compact, lightweight design (20). The rise in various photorefraction systems with different technologies necessitates comparisons with other refraction techniques and established devices in clinical practice and research (11, 14). Despite conventional non-cycloplegic retinoscopy or cycloplegic retinoscopy can be considered as the appropriate measurement techniques that estimates the eye’s spherocylindrical refractive error accurately and precisely in children’s, they can may be impractical in large-scale screening or epidemiologic studies. The scalability of photorefraction screening programs can help reach underserved populations and ensure more children receive necessary vision care. PlusoptiX photorefractors has proven effective in large-scale screening programs, balancing accuracy, speed, and ease of use, are reasonably accurate compared to cycloplegic autorefraction, and shows high sensitivity and specificity for detecting significant refractive errors, with values often ranging between 70-90% (21, 22).

Hence, it becames important to compare new vision screening methods with the PlusoptiX, based on its widespread acceptance and use in vision screening programs (8, 9, 11). The PlusoptiX A16 is a tabletop version of the photorefraction system, similar to the portable systems such as the A12c. According to the manufacturer, the technical characteristics are identical for both devices, except for portability and external design. This endeavour will ensure the precision and efficacy of these new methods in detecting vision problems in children. and provides a practical benchmark against which new devices like the V100 can be evaluated. Additionally, using the PlusoptiX device allows for a direct comparison with existing data and practices, facilitating a more relevant assessment for practitioners who rely on such devices.

In a recent systematic review and meta-analysis non-cycloplegic PlusoptiX was considered the most suitable device for estimating refractive error in young children with low to moderate levels of hyperopia (23). In the present study, was used a version of the PlusoptiX that is not documented in the current literature. However, a study found both PlusoptiX models (S04 and A09) performed equally well in evaluating children.PlusoptiX (24). This suggest the effectiveness of the model we used to be similar to other PlusoptiX models.

Previous studies comparing PlusoptiX to other photorefraction systems have shown PlusoptiX is generally more accurate. A study comparing AI Optic device that resembles the PlusoptiX in form and configuration, PlusoptiXshowed a mean difference in spherical refractive error of -0.10D and that AI Optic does not yet achieve the high performance of the PlusoptiX in terms of targeting uniform amblyopia risk factor guidelines or estimates of sphero-cylinder refractive error (25). Another study comparing PlusoptiX S09 and Spot Vision, finding a mean difference of +0.10D PlusoptiX (26). while, Taberik et al. (21) reported a mean difference of 0.23 D with PlusoptiX A12 compared to Spot Vision ScreenerPlusoptiXwith the PlusoptiX A12 values being more hyperopic (27).

Inter- and intra-subject variability in photorefraction and screening among children are crucial factors affecting the accuracy of refraction measurements (28). Studies have shown that the calibration of luminance slopes in the pupil plays a significant role in determining refractive estimates (29, 30). Ethnic differences have also been highlighted, with the need for ethnicity-specific calibration factors to improve accuracy in photorefraction measurements (29, 30). Additionally, the use of devices like the PlusoptiX screener has been found to be valuable and reproducible in children, despite a tendency for myopic shift at higher refractive errors (31). Enhancements in photorefraction systems and analysis have the potential to correct intersubject variability, making these methods more reliable for pediatric vision screening (28).

Given that the photorefraction luminance profile reflects the underlying optics of the cornea, the luminance profile of photorefraction does become nonlinear in disease that affect the cornea such as keratoconus (32). Photorefraction, particularly eccentric infrared photorefraction, has shown promise in identifying keratoconus progression by analyzing the nonlinearity in the luminance profile of the cornea (32, 33). Eccentric infrared photorefraction reveals a distinct nonlinear luminance profile in keratoconus patients, contrasting with the linear profiles observed in healthy individuals. This nonlinearity increases with disease severity, providing a reliable metric for differentiation and can be integrated into commercial photorefractors, enhancing their diagnostic capabilities for keratoconus (32).

Additionally. photorefraction aids in identifying keratoconus by simulating personalized eye models, distinguishing KC eyes from normal ones, and enabling ophthalmic instrument development and medical training without extensive human subject involvement (33). While photorefraction is a promising tool, combining photorefraction with advanced imaging and AI techniques (34, 35) can enhances diagnostic accuracy. Current technologies for keratoconus screening, have the disadvantage of being bulky, non-portable, expensive and require highly qualified human resources to operate/interpret them. Photorefraction, by contrast, offers a more accessible and portable alternative. Its non-invasive nature, coupled with the ability to detect subtle corneal irregularities indicative of keratoconus, makes it an attractive option for broader clinical and even community-based screenings.

In the present study, we compared the results of two different photoscreeners, the PlusoptiX A16 and the Vision Screener V100, in a population of school-aged children. The goal of the study was to determine if the results obtained with the Vision Screener V100 are comparable to the PlusoptiX A16. The Vision Screener V100 is a new device that, according to the manufacturer, is based on an artificial intelligence protocol and has not yet been evaluated for its capabilities (13). It is an image display device that provides images perceivable from the area of the eye box. It includes a light source unit, a screen with a micro lens array, a scanning unit, and an optical system and have a sensitivity of 81% and specificity of 94% for screening visual acuity deficits. However there are little information about the performance of the Vision Screener V100. We observed a mean difference of 0.06 ± 0.54 D for the spherical equivalent value (M) between the PlusoptiX A16 and the Vision Screnner V100, with the Vision Screnner underestimating the spherical equivalent values compared to the PlusoptiX A16. Although this value is insignificant in clinical terms, we found a worrying tendency for the Vision Screener V100 to overestimate negatively in cases of myopia and positively in cases of hyperopia (r²=0.036, p=0.030). This suggests that the performance of the Vision Screener V100 is not as reliable as the PlusoptiX A16 for higher levels of refractive errors, regardless of whether it is myopia or hyperopia. Consequently, caution should be exercised when interpreting data obtained the Vision Screener V100 in cases of higher refractive error. Clinically, this translates into potential under-correction of myopia and over-correction of hyperopia, and should always be confirmed with additional testing, such as visual acuity for instance or alternative methods such as non-cycloplegic retinoscopy.

For the astigmatism components J0 and J45, we found that only for the J0 component there are statistically significant differences (mean difference 0.09 ± 0.27D). Although there is a slight underestimation of J0 by the Vision Screener V100 compared to the PlusoptiX A16, there was no observed trend for a systematic increase or decrease in the difference between the two devices. For the J45 component, and despite the mean difference found is not statistically significant, we found a tendency for the Vision Screnner V100 to overestimate the values of this component, which indicates that this equipment has a tendency to indicate the presence of oblique astigmatisms when compared to the PlusoptiX A16. Clinicians should be particularly cautious when using the Vision Screener V100 for patients requiring precise axis determination and consider supplementary testing or corroboration with other diagnostic tools including non-cycloplegic retinoscopy and subjective refraction procedures when possible.

To evaluate the clinical significance of the differences observed between the two equipments, the differences were converted into clinical notation. The agreement between the two devices was then examined by calculating the percentage of subjects in which the values obtained for the sphere, cylinder, and axis fell within a range of ±0.75 for the sphere and cylinder, and ±25° for the axis. When the three conditions (sphere, cylinder, and axis) were considered simultaneously, we found that about 1 in 4 subjects (26.3%) had different results obtained with the two methods.

In summary, the difference in the results obtained with the Vision Screener V100 are identical to those reported in other publications that compare the PlusoptiX with different photo-screeners. However, the values obtained for higher refractive errors (higher myopia and hyperopia) suggest that the Vision Screener V100 should be used with caution in populations with these characteristics. The differences observed in the cylinder axis, are also a factor to be taken into account when using the Vision Screener V100. Given the limited age range studied, the results cannot be extrapolated to other ages where the use of this type of equipment may be more useful.

Due to potential variability in the linear operating range across different ethnicities as well as, variations in intraocular scattering, retinal reflectance, pupil size, and the increased inaccuracy at high refraction, the feasibility of the Vision Screener V100 should be assessed with a bigger dataset and further studies are needed to assess its performance across diverse populations and over a large range of refractive error and alsoagainst conventional cycloplegic and non-cycloplegic retinoscopy.

## Conclusion

Despite generally good agreement with the PlusoptiX A16, the present results highlights specific limitations in the performance of the Vision Screening V100, particularly in its accuracy in measuring spherical equivalents and astigmatism components when compared to PlusoptiX A16. The tendency of the Vision Screening V100 to underestimate myopia and overestimate hyperopia should be carefully considered in clinical practice, especially when dealing with children having high refractive errors or astigmatism. Clinicians should be aware of these limitations and consider supplementary testing methods, especially when dealing with high refractive errors or critical axis determinations. The use of alternative, more reliable methods for confirming cylinder axis measurements is recommended to ensure precise and effective vision correction. This is significant because it underscores the need for cautious interpretation measurements, especially in clinical settings where the detection of precise refractive error measurements and correction are essential to prevent visual development issues, such as amblyopia, in children. Future research should explore ways to mitigate these device-specific limitations and understanding and minimizing inter- and intra-observer variability in photoscreening can further enhance the utility of these screening systems, especially in early childhood vision screening.

## Data Availability

Data supporting this study is available upon request by emailing jorge@fisica.uminho.pt.
